# Enhanced Electrochemical Sensor for Electrocatalytic Glucose Analysis in Orange Juices and Milk by the Integration of the Electron-Withdrawing Substituents on Graphene/Glassy Carbon Electrode

**DOI:** 10.1155/2022/5029036

**Published:** 2022-04-12

**Authors:** Rahadian Zainul, Illyas Md Isa, Siti Nur Akmar Mohd Yazid, Norhayati Hashim, Sharifah Norain Mohd Sharif, Mohamad Idris Saidin, Mohamad Syahrizal Ahmad, M. Si Suyanta, Yulkifli Amir

**Affiliations:** ^1^Department of Chemistry, Faculty of Mathematics and Natural Science, Universitas Negeri Padang, Padang, Indonesia; ^2^Department of Chemistry, Faculty of Science and Mathematics, Universiti Pendidikan Sultan Idris, Tanjong Malim, Malaysia; ^3^Nanotechnology Research Centre, Faculty of Science and Mathematics, Universiti Pendidikan Sultan Idris, Tanjong Malim, Malaysia; ^4^Department of Chemistry Education, Faculty of Mathematics and Natural Science, Yogyakarta State University, Yogyakarta, Indonesia; ^5^Department of Physics, Faculty of Mathematics and Natural Science, Universitas Negeri Padang, Padang, Indonesia

## Abstract

In this work, a novel electrochemical sensor was developed by electron-withdrawing substituent modification of 1-phenyl-3-methyl-4-(4-fluorobenzoyl)-5-pyrazolone on a graphene-modified glassy carbon electrode (HPMpFP-graphene/GCE) for glucose detection. The results of characterizations using a scanning electron microscope, Fourier transform infrared spectroscopy, Raman spectroscopy, and nuclear magnetic resonance spectroscopy showed the successful fabrication of HPMpFP-graphene nanocomposite, which served as an electroactive probe for glucose detection. The electron transfer ability of HPMpFBP-graphene/GCE has been successfully revealed using cyclic voltammetry and electrochemical impedance spectroscopy results. The good electrochemical performance was shown by well-defined peak currents of square wave voltammetry under various parameters, including pH, HPMpFP and graphene composition, and scan rate effect. A high electrochemically evaluated surface area using chronoamperometry suggested that the present glucose detection response was intensified. The chronoamperometry results at a work potential of 0.4 V presented a wide linear range of 1 × 10^3^–90 *µ*M and 88–1 *µ*M with 0.74 *µ*M (S/N = 3) as the detection limit. An acceptable recovery has been revealed in the real sample analysis. The electrochemical sensing behaviour of the composite indicates that it may be a promising candidate for a glucose sensor and it significantly extends the range of applications in the electrochemical field.

## 1. Introduction

Clark and Lyons proposed the use of glucose oxidase entrapped inside a semipermeable dialysis membrane built on an oxygen electrode almost 50 years ago [1]. After that, the employment of enzymes in 1970 by Clark showed that electro-inactive substrates were converted into electroactive compounds [2]. Yellow Springs Instrument Company took over Clark's method and developed the first glucose analyzer based on the detection of hydrogen peroxide using an amperometric method in whole blood samples. In 1975, the first glucose analyzer (Model 23A YSI) was successfully launched [3]. Because of the expensive cost of the platinum electrode, this analyzer was virtually exclusively used in clinical laboratories due to the limited availability of other applications. Self-monitoring of glucose levels in the blood was first introduced in the 1980s, and it was used to determine the quantity of glucose present in the blood at any given time. Exactech was the brand name used by MediSense Inc. when they launched the world's first electrochemical glucose meter, a pen-sized electrode strip, in 1987. An amperometric sensor was used to measure the current generated by a glucose oxidase and ferrocene enzyme electrode strip [4].

Food composition information, including glucose content, is vital for the modern food industry as low-quality foods are associated with increased morbidity, mortality, human suffering, and economic burden [5]. Hence, while maintaining high-quality standards and ensuring product safety, food manufacturers also need to make informed purchasing decisions, as well as their preference for high-quality food products at a reasonable price [6]. Continuous improvement and development of analytical methodologies have been responded to match the end-user compliance with food quality. For example, chromatography and mass spectrometry techniques have made it possible to analyze and quantify the contaminants in food samples. However, these traditional technologies are limited by high costs, time-consuming, costly, and complex preparation stages and the necessity for highly trained personnel. In contemporary research articles, the use of electrochemical sensors in food analysis seems promising.

Numerous articles described the use of the enzymatic electrochemical sensing technique for glucose detection in food and beverage samples [7–9]. Many of them focus on the construction and preparation of the sensors and their use for the determination of glucose in aqueous solutions. Using enzyme electrochemical sensors allows very low detection limits and excellent accuracies. Unfortunately, their effectiveness is restricted by the enzyme's temperature, interference, and moisture sensitivity. The enzyme's nature also makes the enzyme expensive and unstable on the electrode surface [10]. To overcome these bottlenecks, recently, numerous articles have described the use of nonenzymatic sensors in the determination of food safety. It is worth developing nonenzymatic electrochemical sensors that allow glucose oxidation on the electrode surface. Low detection limits, excellent physical and chemical durability, increased electron transfer rate, and biocompatibility are the advantages of nonenzymatic electrochemical sensors [11, 12]. A review by Leong et al. discussed nonenzymatic glucose sensors, their glucose oxidation mechanism, and various promising graphene-based nanocomposite systems, as well as the challenges and future prospects of glucose biosensors [13]. Many materials have been investigated for their potential in the development and performance improvement of nonenzymatic glucose sensors, including graphene, noble metals, transition metal oxides, and composites. Also, Balkourani et al. [14] classified the nonenzymatic or enzymeless graphene-based glucose electrocatalyst synthesis methods that have been followed into the last few years such as direct growth of graphene (or oxides) on metallic substrates, in situ growth of metallic nanoparticles into graphene (or oxide) matrix, laser-induced graphene electrodes, and polymer functionalized graphene (or oxide) electrodes.

Graphene is a 2D sheet of sp^2^ carbon atoms organized in a honeycomb-like form. Graphene has 260 times the surface area of graphite and twice that of a carbon nanotube, making it the most densely packed material known [15, 16]. Increasing the surface area allows for more flaws and electroactive sites, enhancing electrochemical catalytic activity on graphene for electrochemical sensing applications [17]. However, aggregations of graphene diminish its accessible surface area and therefore its adsorption capability. This decreases the in-plane conductivity but improves heterogeneous electron/proton transport consistency. Therefore, the use of additional materials to modify graphene for creating graphene-based composites may increase the electrocatalytic performance of the produced sensor while also preventing aggregation [18, 19].

**Figure 1:**
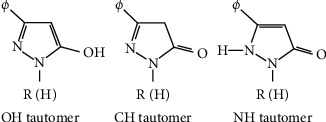
Tautomeric forms of pyrazolones [25].

Pyrazolones, a five-membered lactam ring containing two nitrogen atoms and a ketone group, have been widely studied as starting materials for more sophisticated heterocyclic systems with applications in the pharmaceutical industry. They are also structurally fascinating compounds since they exhibit tautomerism. They exist in three different tautomeric forms (Figure 1), namely 3-pyrazolone, 4-pyrazolone, and 5-pyrazolone [20]. This phenomenon may have an impact on their responsiveness, as well as the synthetic techniques in which they participate and the biological activities of targets because changes in structure lead to changes in attributes [21].

1-phenyl-3-methyl-4-(4-fluorobenzoyl)-5-pyrazolone (HPMpFP) is among the class of *ß*-diketones with a pyrazolone ring where their primary structural feature is attached to a chelating carbonyl group. The compounds have been widely employed in prospective anticancer agent regions [22, 23] and have played a significant role in incorporating coordination chemistry. Profited from the wide use of pyrazolone materials, it is possible to contemplate HPMpFP in the field of electrochemical sensors. Electron-withdrawing substituents such as fluorine in 1,3-*β*-diketone pyrazolone improve the electronic properties of the ligand [24]. As supported by Ghoneim et al. [25], the enol from the OH of the acyl pyrazolones is mildly acidic, making it appropriate for electrochemical sensing applications and has practical importance in polarographic detection of trace metals [26, 27]. Recently, no endeavours have been made to develop the HPMpFP for the improvement of the electrochemical glucose sensor. Prior research has utilized 1-phenyl-3-methyl-4-(2-furoyl)-5-pyrazolone (HPM*α*FP) for electrochemical detection of adenine [28], guanine [29], xanthine [30], thymine [31], oligosaccharides [32], and tyrosine [33].

In this article, HPMpFP was first applied as a modifier to obtain a composite with a graphene-modified glassy carbon electrode (HPMpFP-graphene/GCE), which was tested for electrochemical detection of glucose. The aromaticity of HPMpFP may be adsorbed onto the surfaces of graphene to create the composite [34, 35]. Combining the benefits of these two materials may speed up electron transport, improve electrochemical character, and increase electrode active surface area. The electrochemical detection of glucose in beverage samples has been studied using the HPMpFP-graphene/GCE material for real sample analysis.

## 2. Materials and Methods

### 2.1. Reagents and Materials

Graphite powder (spectroscopically pure reagent), potassium ferricyanide, glucose, N,N-dimethylformamide, sodium carbonate, dipotassium hydrogen phosphate (K_2_HPO), and potassium dihydrogen phosphate (KH_2_PO_4_) were obtained from Merck (Germany). Sigma-Aldrich (USA) provided diethyl ether, potassium dioxide, and 4-fluorobenzoyl chloride. Analytical grade chemicals were utilized without any additional purification being required. K_2_HPO_4_ and KH_2_PO_4_ were used to make 0.1 M phosphate buffer solution (PBS), and H_2_SO_4_ and NaOH were added dropwise to alter pH. Prior to usage, glucose solutions were introduced to PBS in a fresh manner. Distilled deionized water from EASY Pure LF, Barnstead (USA), has been used for the preparation of the solutions to provide the highest quality results.

### 2.2. Apparatus

A three-electrode configuration with a potentiostat/galvanostat/ZRA 3000 was used for voltammetric investigations (Gamry, USA). There was an Ag/AgCl reference electrode and an auxiliary electrode utilized, where the working electrodes were either bare or modified GCEs. The electrochemical studies were carried out in an electrolyte that had been nitrogen-purged prior to the experiment. Field emission scanning electron microscopy was utilized to study the material's morphology (FESEM, Hitachi SU 8020 UHR, Japan). The Agilent Cary 620/670 Series equipment was used in a Fourier transform infrared (FTIR) spectroscopy experiment that used the KBr disc method with an in-band wavelength range of 400 cm^−1^ to 4000 cm^−1^. inVia Raman spectroscopy was used to capture the Raman spectra, which were stimulated by a 514 nm Argon laser. Nuclear magnetic resonance spectroscopy was used to get ^1^H and ^13^C NMR spectra (NMR, JNM-ECX 500 JEOL, Japan).

### 2.3. Synthesis of HPMpFP Complex

**Scheme 1:**
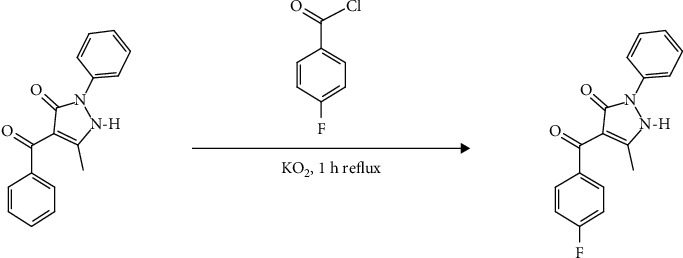
Synthesis of the HPMpFP structure.

The HPMpFP complex has been prepared using the benzoylation process according to a previous study with slight modifications [36]. In round bottom flasks, 180 mL of diethyl ether was added initially, followed by 30 g of 1-phenyl-3-methyl-4-benzoyl-5-pyrazolone, which was then added to the flasks after that. The mixture was then heated to the point of dissolving all the mixed compounds. The flask was filled with 24 g of potassium dioxide and was rapidly stirred. Following the heating procedure, 20 mL of 4-fluorobenzoyl chloride was added dropwise to the mixture and refluxed for another 1 hour. The mixture was then put into acidic water (3 M, 300 mL) to produce crystalline solids. A whitish-grey crystalline powder was recovered and purified after a few days via a series of recrystallization processes that were carried out using a methanol-water mixture. The finished product was dried at room temperature and labelled HPMpFP. The synthesis of HPMpFP is shown in Scheme 1.

### 2.4. Preparation of HPMpFP-Graphene Composite

Graphene was produced using reduction process, which included the use of sodium carbonate as a reducing agent [37]. Typically, 2 mL (0.05 mg/mL) of graphene powder was disseminated in 20 mL of N, N-dimethylformamide to get a uniform dispersibility producing 2.45 × 10^−5^ M of graphene solution. To improve performance, the graphene and HPMpFP compositions in the solution were adjusted in control tests. About 0.05 mg/mL of HPMpFP complex solution was added to the aforementioned solution in various amounts (0.5, 0.8, 1.0, 3.0, and 5.0 percent v/v) and sonicated for 30 minutes.

### 2.5. Preparation of the Sensor

Before casting, the GCE surface was thoroughly polished using a pure powder made from alumina with a particle size of 0.05 mm, which was used to achieve the desired finish, followed by deionized water and ethanol for 5 minutes to produce a mirror-like surface. The electrode may then dry at room temperature under the blowing of nitrogen. The HPMpFP/graphene-GCE was created by casting HPMpFP-graphene suspension with different compositions onto the GCE surface after it had been cleaned. The modified electrode was then tested. The reactions of the square wave voltammetry (SWV) were recorded, and the optimum composition was determined (1.0% v/v of 0.05 mg/mL HPMpFP in 0.05 mg/mL graphene suspension), which was then chosen for the following studies aimed at detecting glucose levels. A beaker was placed over the electrode, allowing the water to evaporate slowly over time, lifting a consistent coating created on the electrode surface. The beaker was set up so that the water would evaporate over time, lifting a consistent coating created on the surface of the electrode. It was the same manufacturing technique that was used to produce unmodified graphene and GCE, except the HPMpFP complex that was not added to the solution during fabrication.

### 2.6. Real Sample Analysis and Validity Test

Orange juice and milk were purchased from a local market in Perak, Malaysia. Real sample analysis was performed on these real samples (0.5 mL in 20 mL of 0.1 M PBS) to determine the concentration of glucose. An equal volume of glucose solution was added to each sample and was examined under optimum circumstances to get the average. The *t*-test was conducted for validity analysis of the prepared sensor compared with a commercial glucometer.

## 3. Results and Discussion

### 3.1. Characterizations

**Figure 2:**
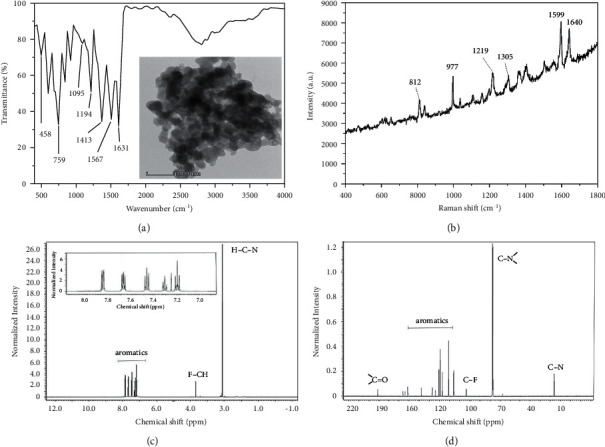
(a) FTIR spectrum (inset: TEM micrograph), (b) Raman spectrum, (c) 1H, and (d) 13C NMR spectra of the HPMpFP complex.

The important infrared frequencies of HPMpFP are presented in Figure 2(a). Its successful formation was evident in the presence of a wideband at 3152 cm^−1^. This might be due to the vibration of –OH stretching in the HPMpFP molecule, which was hydrogen-bonded to the molecule's carbonyl group. The peaks of carbonyl (C=O) and pyrazolone ring were present at 1631 cm^−1^ and 1413 cm^−1^, respectively. The peak at 1567 cm^−1^ was integrated into the C–N stretching of the phenyl ring, and the C–F stretching was presented by the peak of 1194 cm^−1^. In the complex, the C–H stretching and phenyl ring stretching were disclosed by the peak at 1095 cm^−1^, whereas the C–H bending of the phenyl ring in the complex was shown by the vibrational frequency modes between 759 and 458 cm^−1^ [38, 39]. This infrared evidence reveals that this compound exists in the enol form. The microscopic image of HPMpFP depicted in the inset of Figure 2(a) displays a spherical shape of the HPMpFP complex.

As shown in Figure 2(b), the Raman spectra of the HPMpFP complex were obtained in the frequency range of 400 to 1800 cm^−1^, with the peak at 400 cm^−1^. When the free carbonyl group in the benzoyl group was stretched, it caused a peak at 1640 cm^−1^, which was caused by the stretching vibration. The stretching vibration of the intermolecular hydrogen-bonded carbonyl group in the pyrazolone ring was attributed to the peak at 1599 cm^−1^ in this experiment [40]. Furthermore, the peaks at about 977 to 1209 cm^−1^ were shown to be integrated with the ring-breathing vibration of the phenyl ring system. The peak at 812 cm^−1^ was allocated to asymmetrical vibration involving the fluoro group.

In ^1^H NMR spectra (Figure 2(c)), the distinct peaks at 7.8712 ppm to 7.1246 were assigned to the resonance of aromatic structures, such as C=O and the phenyl ring. The peak that appeared at a chemical shift of 3.8301 ppm was the resonance of F–CH, and the peak at 3.1290 was the resonance of H–C–N. The ^13^C NMR spectra (Figure 2(d)) showed a peak in chemical shift of 192.1513 ppm, which was assigned to the resonance for C=O and some peaks in chemical shift of 160.9213 ppm to 113.2519 ppm, which were assigned to the resonances for aromatic phenyl structures. Furthermore, several peaks appeared at chemical shifts of 103.5114 ppm, 78.3125 ppm, and 14.1151 ppm, which were assigned to the C–F, C–N, and C–H assignments. Therefore, it can be concluded that the HPMpFP synthesized was pure and these characterizations were in agreement with the HPMpFP.

### 3.2. Electrochemical Performance of HPMpFP-Graphene/GCE

**Figure 3:**
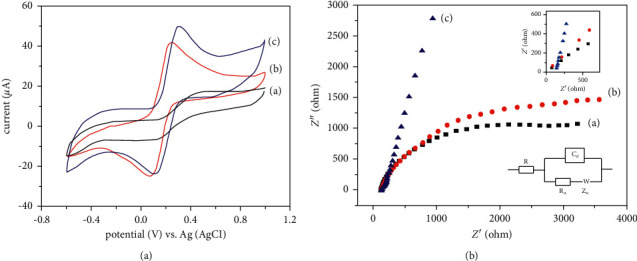
In 4 mM K3 [Fe(CN)6] containing 1.0 M KCl, the CV curves of the unmodified GCE (a), graphene/GCE (b), and HPMpFP-graphene/GCE (c) were shown (a), as were the Nyquist plots of the unmodified GCE (a), graphene/GCE (b), and HPMpFP-graphene/GCE (c) (the Randles equivalent system circuit is shown in the inset) (b).

Electrochemical studies examined the electrochemical characteristics of HPMpFP-graphene/GCE, which was studied using cyclic voltammetry (CV) and electrochemical impedance spectroscopy (EIS) with the supporting electrolyte potassium ferricyanide (K_3_ [Fe(CN)_6_]). As shown in Figure 3(a), the results of CV curves of the as-prepared bare GCE (a), graphene/GCE (b), and HPMpFP-graphene/GCE (c) were obtained by scanning the potential from −0.60 to +1.20 V at a scan rate of 100 mV/s. The observations of the curvature of GCE before it was modified revealed a redox peak with cathodic and anodic peak potentials of 0.072 V and 0.439 V, respectively, with a peak separation (Δ*E*_p__=_*E*_pc_ − *E*_pa_) of 0.367 V. The redox peak potentials were raised by 0.158 V and 0.139 V with the addition of graphene and HPMpFBP-graphene composite. For bare GCE, the current ratio of the oxidation peaks to the corresponding reduction peaks (*i*_pa_/*i*_pc_) was 1.04. For graphene/GCE, the current ratio was 0.93, and for HPMpFP-graphene/GCE, the current ratio was 0.97. As the ratios were close to one, these results were due to a reversible voltammogram (*i*_pa_/*i*_pc_ = 1) [41].

As previously mentioned, Table 1 indicates that the peak current execution of HPMpFP-graphene/GCE was superior to graphene/GCE and bare GCE. Even after the modification procedure, the background current on the HPMpFBP-graphene/GCE is much greater than the CV curve at unmodified GCE and graphene/GCE, indicating that the electrode has a large surface area. This was additionally in acceptable concurrence with the electrochemical impedance spectroscopy (EIS) results of this study.

The EIS was carried out to take into consideration the electrochemical characteristics of the modified electrodes via the use of a redox probe of 4 mM K_3_ [Fe(CN)_6_] with 1.0 M KCl. The direct part of the semicircle depicts the diffusion process, while the whole semicircle represents an active control barrier that acts to reduce the flow of electrons at the electrode contact, which is also known as electron transfer resistance (*R*_ct_) [42]. The EIS curve of K_3_ [Fe(CN)_6_] at the bare GCE showed a small semicircle in the high-frequency area and is a straight line in the low-frequency region, as shown in Figure 3(b). In the meantime, the breadth of the graphene/GCE semicircle has shrunk due to its high stability and electrical conductivity. Simultaneously, the semicircle was almost invisible at the HPMpFP-graphene/GCE, indicating the electrode's low *R*_ct_. The Randles equal circuit viable with the impedance spectrum is shown in the inset of Figure 3(b). This circuit explains the resistance of the electrolyte solution, double-layer capacitance, and the Warburg impedance by referring to symbols such as *R*_s_, *C*_dl_, and *Z*_w_.

Using the Randles equivalent electrical circuit, it was possible to calculate the value of *R*_ct_ for HPMpFBP-graphene/GCE, which was found to be lower (20.86) than graphene/GCE (43.14) and bare GCE (80.97). The electrode with the lowest *R*_ct_ value has the best conductivity. It tends to be summed up that the arrangement of *R*_ct_ on the electrodes expanded in the request for HPMpFP-graphene/GCE < graphene/GCE < unmodified GCE. The astounding conductivity of HPMpFP-graphene/GCE demonstrates the extraordinary electron move capacity. In the development of electrochemical sensors, this is an important factor to consider. The electron move rate steady (*k*_app_) values obtained from Equation (1) also support this. When K_3_ [Fe(CN)_6_] undergoes a redox reaction, the amount of moles of electrons transported (*n*) is equal to one.

(1) $$\begin{array}{c} k_{\text{app}}=\frac{RT}{nF2RctCA}. \end{array}$$

From Table 1, it can be shown that the *k*_app_ value achieved for HPMpFP-graphene/GCE was greater than the corresponding values found for graphene/GCE and bare GCE. Consequently, it can be concluded that the HPMpFP-graphene/GCE, which benefited from the synergistic impact of the HPMpFP complex and the graphene composite, has produced excellent electrochemical sensor characteristics as a result of its use.

### 3.3. Responses to Glucose on an Electrochemical Reaction

**Figure 4:**
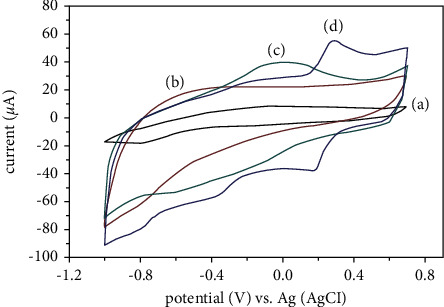
CVs of the HPMpFP-graphene/GCE in the absence of glucose (a) and the presence of 0.1 mM of glucose in 0.1 M PBS for bare GCE (b), graphene/GCE (c), and HPMpFP-graphene/GCE (d).

The electrochemical effectiveness of various prepared electrodes was determined using CV with the presence and absence of 0.1 mM glucose, which was tested in 0.1 M PBS (pH 7) electrolyte solution. The experiments shown in Figure 4 used the following materials: unmodified GCE, graphene/GCE, and HPMpFP-graphene/GCE. In the absence of glucose, HPMpFP-graphene/GCE could not detect any CV signal for the no oxidation or glucose reduction (curve a) approach. Bare GCE (curve b) still had no peak current, suggesting poor sensitivity. The curve d for HPMpFP-graphene/GCE achieved the greatest peak current response to glucose detection, whereas the curve c for graphene/GCE had a greater CV peak current.

This modified electrode's electrocatalytic reaction to glucose is beneficial for the following reasons. First, the outstanding electrical conductivity of graphene helps increase the glucose-attracting ability of the resulting electrode, thereby increasing its large surface area. Second, graphene is not only integrated with HPMpFP aromaticity that is adsorbed on graphene surfaces through *π*-*π* strength and hydrogen bonding to form the composite, but it is also used to construct a conductive interconnivance network that can prevent graphene coagulation and increase surface area. Third, introducing electron-withdrawing substituents such as fluorine into the 1,3-*β*-diketone position of the HPMpFP may improve the ligand's electrical characteristics, thereby enhancing glucose detection at the modified electrode surface.

### 3.4. pH Impact on the Effectiveness of the Electrode

**Figure 5:**
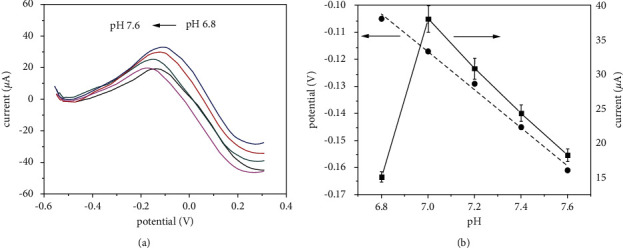
SWVs of the HPMpFP-graphene/GCE in the presence of 0.1 mM glucose in 0.1 M PBS at various pH levels: 6.8, 7.0, 7.2, 7.4, and 7.6 are shown in (a), and the connection between various pH values and peak potentials, as well as the connection between various pH values and peak currents, is presented in (b).

Using SWV, the impact of pH (6.8 to 7.6) on the peak glucose potential at the HPMpFP-graphene/GCE in 0.1 M PBS was investigated (Figure 5(a)). The results showed that the maximum potential changes adversely with increased pH, which indicates that the proton participates directly in the electrochemical process [43]. As the pH value rose from 6.8 to 7.0, the glucose oxidation peak current increased, and as the pH went higher than 7.0, the glucose oxidation peak current dropped. The pH value of 7.0 of the PBS electrolytes was determined to be the optimal experimental parameter for the electrochemical detection technique that will be used next. Additionally, in Figure 5(b), the connection between peak potentials (*E*_p_) and pH levels is shown. It is possible to describe this connection mathematically using the linear regression equation, which may be expressed as follows:

(2) $$\begin{array}{c} E_{\text{p}}\left(V\right)=-0.062\text{ pH}+0.373\left(RR=0.992\right). \end{array}$$

**Scheme 2:**
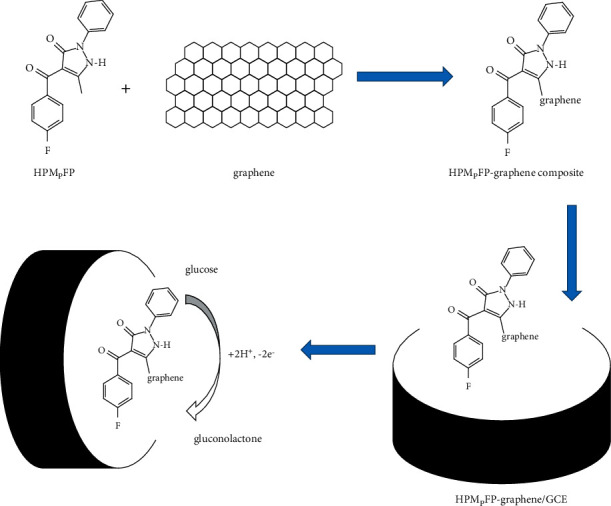
Possible reaction mechanism for HPMpFP-graphene/GCE.

The slope was neared to the theoretical value of 58.6 mV/pH. This indicates that during the glucose redox process the total number of electrons and protons taking part was identical [44]. Following the process shown in Scheme 2, it is still expected that the reaction product of glucose electrooxidation with the HPMpFP-graphene composite would result in the formation of gluconolactone. The high surface-to-volume ratio of the composite, as well as its excellent electron transport route, significantly aided the electrocatalytic process. As a result, the HPMpFP-graphene composite has the potential to be a superior glucose sensor electrode.

### 3.5. Amount of HPMpFP-Graphene Composite Material Cast on GCE

**Figure 6:**
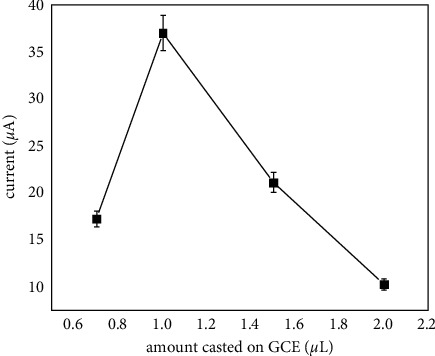
SWVs of HPMpFP-graphene/GCE with different amounts of HPMpFP-graphene composite material cast on GCE.

A droplet of HPMpFP-graphene composite was cast on the GCE surface based on the electrode preparation stage, and a thin layer of the sample was left behind on the GCE due to solvent evaporation. Therefore, by changing the amount of dispersion cast on the GCE surface, the thickness of the HPMpFP-graphene composite layer could be altered. The SWVs of prepared electrodes with different quantities of composite HPMpFP-graphene cast on GCE are shown in Figure 6. It was found that increasing the dispersion volume from 0.75 to 1.00 *µ*L resulted in a substantial rise in peak current, while increasing the dispersion volume further to 2.0 µL resulted in a considerable decrease in peak current. This is likely to become more difficult due to the excessive thickness of sample dispersion and electrode surface diffusion. Therefore, 1.0 *μ*L of HPMpFP-graphene composite dispersion was selected for electrode preparation in this study.

### 3.6. The Impact of Scan Rate

**Figure 7:**
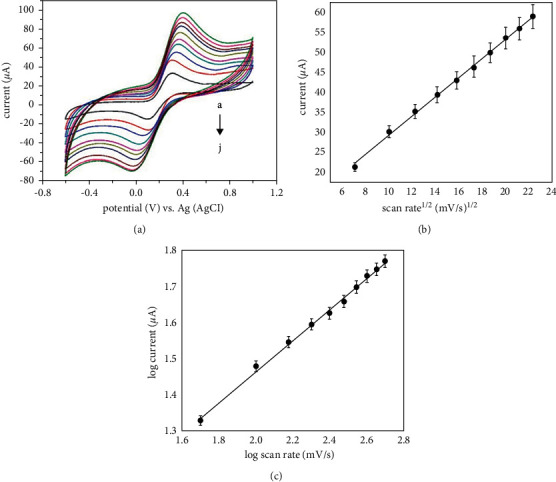
CV of the HPMpFP-graphene/GCE towards glucose detection at various scan rates (20 to 220 mV/s) (a), the plot of peak current against the square root of scan rate (b), and the plot of log peak current versus the log of scan rate (c).

Figure 7(a) depicts the scan rate impact on the CV peak currents of HPMpFP-graphene/GCE when the scan rate is increased. This parameter was measured using an electrolyte containing 0.1 mM glucose in 0.1 M PBS (pH 7.0) in the range of −0.6 to 1.0 mV/s and a pH of 7.0. The redox peak currents progressively rose as the scan rate increased from 20 to 220 mV/s, while the anodic (*E*_pa_) and cathodic peak (*E*_pc_) potentials shifted positive and negative, respectively. The values of peak separation (Δ*E*_p_ = *E*_pc_–*E*_pa_) were calculated to be between 0.215 and 0.455 V at different scan rates, which illustrates the sluggish electron transport kinetics. The difference between the anodic and cathodic peaks in these redox reactions is related to ion transport resistance in these processes [45]. The reliance on the square root of the scan rate (*v*^1/2^) of the cathodic peak current (*i*_pc_) is an important diagnostic criterion for determining the type of reaction mechanism. The figure shows a linear relationship between the glucose *i*_pc_ and the *v*^1/2^. This suggests that the diffusion-controlled current system has been used to detect glucose at HPMpFP-graphene/GCE. The oxidation peak currents followed the linear regression equation:

(3) $$\begin{array}{c} i_{\text{pc}}=2.39v^{1/2}+5.32\left(RR=0.998\right). \end{array}$$

A log plot was used to further validate the reversibility of the sensor that had been constructed (Figure 7(c)). This correlation was revealed by plotting a linear relationship between the logarithm of cathodic peak current (log *i*_pc_) and the logarithm of scan rate, which revealed that the linear regression equation could be expressed as follows:

(4) $$\begin{array}{c} \log\;i_{pc}=0.43\;\log\;\nu +0.61\left(RR=0.996\right). \end{array}$$

Using this method, a slope value of 0.43 was achieved, which is less than the theoretical value of 1.0 that was anticipated to occur at the surface of the electrode. Furthermore, this supports the notion that, following the adsorption of the glucose molecules, the entire electrode process is primarily controlled by diffusion [46] because the lower actual slope (0.43) than the predicted value may be due to glucose molecules' partial participation in the HPMpFP-graphene/GCE response.

### 3.7. Electrochemically Efficient Surface Areas Using Chronocoulometry

It has been found that the electrochemically efficient surface areas of unmodified GCE and HPMpFP-graphene/GCE were measured utilizing the chronocoulometric method, which specifies the charge-time dependency for linear diffusion control, namely the Anson equation [47].

(5) $$\begin{array}{c} Q=2nFA_{\text{real}}C\frac{\sqrt{Dt}}{\sqrt{\pi }}+Q_{\text{dl}}. \end{array}$$

The surface area of the working electrode is indicated by *A*, the diffusion coefficient is denoted by *D*, the concentration of the substrate is denoted by *C*, the Faradic charge is denoted by *Q*_ads_, and the double-layer charge is marked by *Q*_dl_. At 25°C, the standard diffusion coefficient (*D*) of K_3_ [Fe(CN)_6_] is 7.6 × 10^−6^ cm^2^/s, and the electron number is one [48].

**Figure 8:**
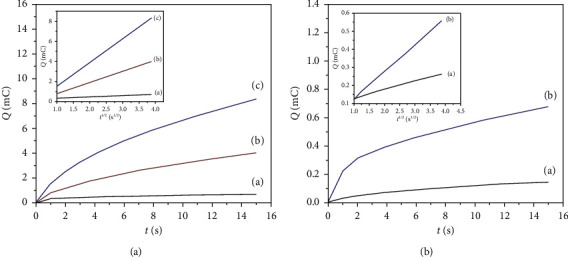
Plots of Q-t curves (A) for unmodified GCE (a), graphene/GCE (b), and HPMpFP-graphene/GCE (c) in 4 mM K3 [Fe(CN)6] containing 0.1 M KCl (inset: plots of Q-t1/2 curves for each electrode, respectively); plots of Q-t curves (B) for the HPMpFP/graphene-modified GCE in 0.1 M PBS in the absence (a) and presence (b) of 0.1 mM glucose, respectively (inset: the plot of Q-t1/2 curves on HPMpFP-graphene/GCE).

The slope of the linear connection between *Q* and *t*^1/2^ was used to derive the following equation for unmodified GCE, graphene/GCE, and HPMpFP-graphene/GCE, which was found to follow with the findings presented in Figure 8(a) for all three electrodes.

(6) $$\begin{array}{c} Q=0.124\times 10^{-3}t^{1/2}-0.213\times 10^{-3},\\ Q=1.126\times 10^{-3}t^{1/2}-0.374\times 10^{-3},\\ Q=2.365\times 10^{-3}t^{1/2}-0.823\times 10^{-3}. \end{array}$$

According to the slopes of 0.124 × 10^−3^ mC/s^1/2^ (bare GCE), 1.126 × 10^−3^ mC/s^1/2^ (graphene/GCE), and 2.365 × 10^−3^ mC/s^1/2^ (HPMpFP-graphene/GCE), *A* was calculated to be 1.12 × 10^−4^ cm^2^, 1.01 × 10^−3^ cm^2^, and 6.13 × 10^−3^ cm^2^ for unmodified GCE, graphene/GCE, and HPMpFP-graphene/GCE, respectively. In addition to increasing the number of electrode reaction sites, increasing the adsorption capacity, and intensifying the current response of glucose, the increased electrode surface may also improve the sensitivity and detection limit of this sensor.

In addition, HPMpFP-graphene/GCE was also subjected to chronocoulometry analysis in the absence and presence of 0.1 mM glucose for 30 minutes. When *Q* was plotted against time *t*^1/2^ (Figure 8(b)), it revealed a linear connection with the following equation, where the slope was 1.471 × 10^−3^ mC/s^1/2^ and the intercept (*Q*_ads_) was 0.098 × 10^−3^ mC.

(7) $$\begin{array}{c} Q=1.471\times 10^{-3}t^{1/2}-0.098\times 10^{-3}. \end{array}$$

When the background is subtracted from the linear connection between *Q* and *t*^1/2^, the intercept of the linear relationship corresponds to *Q*_ads_. As *n* = 2, *A* = 5.31 × 10^−4^ cm^2^, and C = 0.1 mM, the *D* was estimated to be 1.6194 × 10^−4^ cm^2^/s. The adsorption capacity (Γ_s_) of glucose at the HPMpFP-graphene/GCE was calculated to be 9.58 × 10^−7^ mol cm^−2^, following with equation:

(8) $$\begin{array}{c} Q_{\text{ads}}=nFA{Г}_{s}. \end{array}$$

### 3.8. The Influence of Potential Based on the Amperometric Response

**Figure 9:**
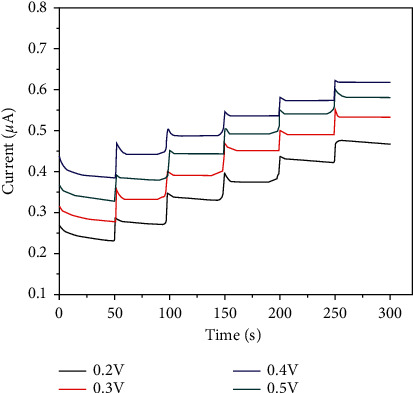
Amperometric response of HPMpFP-graphene/GCE with successive addition of 5 mL 0.1 mM glucose to 0.1 M PBS at different applied potentials (from 0.2 V to 0.5).

The dependence of the potential applied to the amperometric signal of HPMpFBP-graphene/GCE is shown in Figure 9 with the successive addition of 5 mL 0.1 mM glucose to 0.1 M PBS at the different potentials applied. After increasing the applied voltage from 0.2 to 0.4 V, the response of the prepared sensor rose quickly for a short period before decreasing when the potential was raised to 0.5 V. The increasing reaction with applied potential can be attributed to the increased driving force of HPMpFP-graphene electrooxidation of glucose. As a result, the applied potential was determined to be 0.4 V in the following experiments.

### 3.9. Linearity and Detection Limit

**Figure 10:**
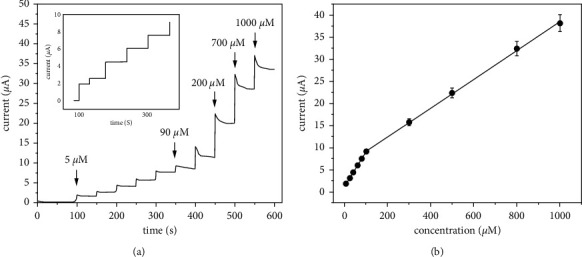
Chronoamperometric response of HPMpFP-graphene/GCE with sequential addition of different glucose concentrations (a) and plot of current response against glucose concentration at HPMpFP-graphene/GCE (inset shows the current responses between 5 and 90 µM concentrations of glucose) and (b) the plot of current response vs. glucose concentration.

Rather than using SWV or CV, the chronoamperometry approach was utilized in this research for quantitative glucose analysis since it has better sensitivity and resolution than other methods. After the first administration, a 50-second interval was given among the following doses, and the glucose concentration was varied from 5 to 1000 *µ*M. As shown in Figure 10(a), the concentration of glucose was first raised in steps of 5 *µ*M, then 15 *µ*M, and finally 25 *µ*M, before being stopped. Later, the increments were raised by 45 *µ*M at a time, until the concentration reached 90 *µ*M. Finally, 100 *µ*M increments were made until the final 1000 *µ*M step-in addition.

Meanwhile, Figure 10(b) revealed the current responses amplified at the HPMpFBP-graphene/GCE with an increased glucose concentration from 5 to 1000 *μ*M. There were two linear regions in this range (Figure 10(b)), where the current response grew quickly as the glucose concentration rose in the first linear area among 5 and 90 µM, and the linear regression equation was depicted as I (*μ*A) = 0.07 + 1.46 C (*R*^2^ = 0.996) with a sensitivity of 11.42 *μ*A/mM/cm^2^. Meanwhile, the current continued to rise in the second area, starting at 88 µM, when the linear regression equation was found to be I (*μ*A) = 0.032 + 6.074 C (*R*^2^ = 0.998) and the sensitivity was measured as 5.22 *μ*A/mM/cm^2^. It was estimated that the detection limit was 0.74 *μ*M (S/N = 3). Compared with other carbon-based materials reported in the literature (see Table 2) specifically on glucose sensing in food and beverages, these findings were substantial and improved, clearly demonstrating that the produced nonenzymatic electrode has a broad linear range, a lower detection limit, and a greater sensitivity than previously reported. Even though Ref. [7] presented a lower LOD than this work, their study used enzyme. Using enzyme electrochemical sensors allows very low detection limits and excellent accuracies. Unfortunately, their effectiveness is restricted by the enzyme's temperature, interference, and moisture sensitivity. The enzyme's nature also makes the enzyme expensive and unstable on the electrode surface [10]. For these reasons, it is worth developing nonenzymatic electrochemical sensors, which allow glucose oxidation on the electrode surface to overcome these drawbacks.

### 3.10. Interference Study

**Figure 11:**
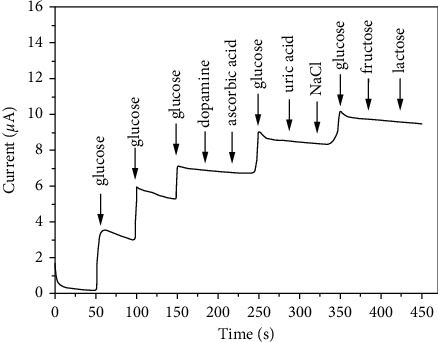
HPMpFP-graphene/GCE interference analyses.

The amperometric response of HPMpFP-graphene/GCE at 0.4 V with 0.01 mM interfering reagent concentration and 0.1 mM glucose present in 0.1 M PBS solution indicated that the addition of dopamine and ascorbic acid had no amperometric effect (Figure 11). Further investigation revealed that when 0.1 mM glucose was added to the PBS solution after the addition of the interfering reagents, it caused the amperometric response to increase in the same manner as before the electrode was indicated. This finding exhibited good glucose selectivity and was unaffected by other interfering reagents such as uric acid, sodium chloride, fructose, and lactose.

### 3.11. Reproducibility, Repeatability, and Stability

**Figure 12:**
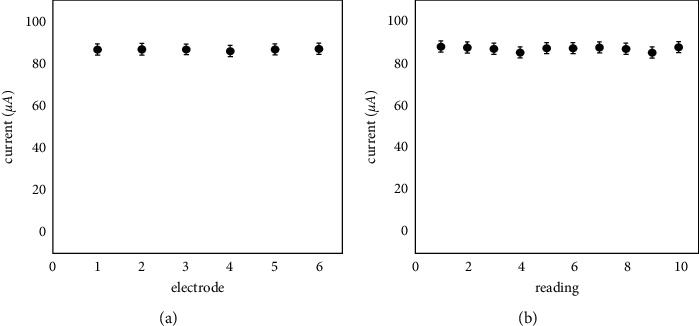
(a) Reproducibility and (b) repeatability of HPMpFP-graphene/GCE.

The performance of the electrode in terms of reproducibility, repeatability, and stability are all important characteristics. They were investigated by measuring the responses of the HPMpFP-graphene/GCE in the presence of 0.1 mM glucose in 0.1 M PBS (pH 7). The reproducibility was tested using six identically produced electrodes, and the relative standard deviation (RSD) was determined to be 2.52 ± 0.51% (Figure 12(a)). Ten consecutive measurements were taken with the same modified electrode, and the repeatability was found to be 2.13 ± 0.72% when the RSD was calculated (Figure 12(b)). These results show that the HPMpFP-graphene/GCE was stable and that it could be used for glucose monitoring regularly.

**Figure 13:**
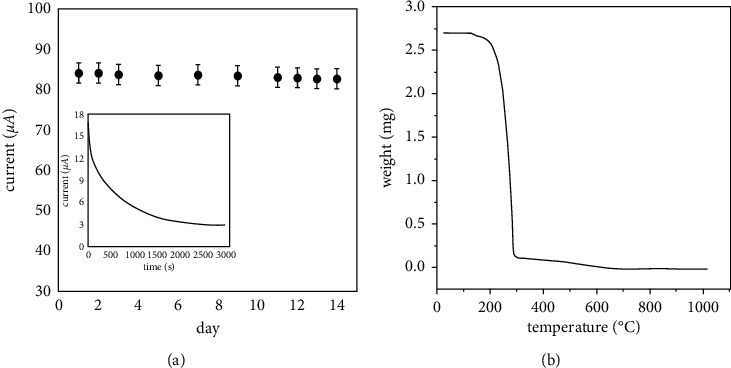
(a) HPMpFP-graphene/GCE was subjected to a stability test and a chronoamperogram for 0.1 mM glucose in 0.1 M PBS at +0.4 V for 3000s operating time. The results showed that the HPMpFP-graphene/GCE was stable for 3000s (see inset) and (b) TGA analysis.

The stability of the as-prepared sensor was determined by monitoring the current response to glucose detection after two weeks of operation (Figure 13(a)). The current was observed to preserve around 90% of its original response after two weeks of storage, suggesting the high stability of HPMpFP-graphene/GCE. After 3000 s of continuous measurement, the amperometric response current of the constructed electrode dropped to less than 8% (see inset Figure 13(a)), showing long-term stability in the detection of glucose. Accordingly, the TGA curve was applied to monitor the changing process in the view of weight loss. As shown in Figure 13(b), at an initial stage of 35–220°C, the weight loss could be mostly attributed to the moisture evaporation. The major weight loss occurred at the second stage 220–310°C and a final weight loss at 310–800°C as the result of degradation and decomposition of the HPMpFP-graphene composite.

### 3.12. Real Sample Analysis and Validity Test

To show the viability of nonenzymatic HPMpFP-graphene/GCE measurements in real-world glucose, this precise sensor was used to detect glucose concentrations in orange juice and milk samples.  Table 3 shows the findings obtained using the conventional method of addition, in which each sample was monitored under optimal circumstances for the mean of three determinations. The results indicated that the recoveries varied from 90.29 ± 3 to 103.16 ± 2 and proved the efficiency and the reliability of this approach. It was also necessary to conduct a *t*-test analysis to determine whether any of the findings obtained in the presence of a known amount of glucose added to the PBS solution were legitimate. As indicated in Table 3, not all of the values acquired by the sensor in its as-prepared state were compatible with the values obtained by the glucometer utilized in the clinical trials. For *p* < 0.05, there was no statistically significant difference, while for *p* > 0.05, there was a statistically significant difference. These findings suggest that nonenzymatic HPMpFP-graphene composite sensors may be employed in glucose determination in beverages.

## 4. Conclusions

In summary, the fabrication of HPMpFP-graphene/GCE sensors for selective glucose detection was described in this study. Under optimal conditions, the peak current for glucose detection on HPMpFP-graphene/GCE was significantly higher than the peak currents on graphene/GCE and unmodified GCE. As a result, the integration of the greater surface area and unique graphene conductivity of the composite with the HPMpFP complex significantly improved the performance of the sensor. Diffusion-controlled redox processes of glucose were shown to be more common in the presence of HPMpFP-graphene/GCE. Low detection limits for glucose and a wide concentration range for glucose were shown by the HPMpFP-graphene/GCE, demonstrating good selectivity and sensitivity towards glucose. The HPMpFP-graphene/GCE was effectively utilized to detect glucose in certain orange juice and milk samples, with a recovery rate ranging from 90 to 103%.

## Figures and Tables

**Table 1:** EIS of the electrode.

Electrode	*i*_pc_ (*µ*A)	*i*_pa_ (*µ*A)	*R*_ct_ (Ω cm^2^)	*k*_app_/cms^−1^
Bare GCE	2.17	2.25	80.97	5.02 × 10^−3^
Graphene/GCE	15.42	14.34	43.14	9.30 × 10^−3^
HPMpFP-graphene/GCE	28.13	27.39	20.86	16.41 × 10^−3^

**Table 2:** Compared with previous electrodes, the performance of the HPMpFP-graphene/GCE in measuring glucose concentrations was shown to be improved.

Electrode	Sensitivity (*μ*A/mM/cm^2^)	Linear range (µmol L^−1^)	LOD (µmol L^−1^)	Sample	Ref.
^a^PEDOT/^b^PAA/^c^GOD and PEDOT/^d^AA/GOD	274 ± 7 and 257 ± 10	30 × 10^3^–980 and 30 × 10^3^–1860	0.29 and 0.56	Grape juice and honey	[7]
^e^PAA-^f^VS-PANI/^g^GPL-FePc/^h^GOx-^i^CH	18.11	20 × 10^3^–1 × 10^3^	6.40	Fruit juice and human serum	[49]
^j^Ag-PANI/^k^rGO	2.7664	50–0.1	0.79	Orange juice, apple juice, mango juice, coke, and milk	[50]
Carbon-supported ^l^PdCoAg	4156.34	350–5	3.00	Energy drink, fruit juice, and carbonated beverages	[51]
HPMpFP-graphene/GCE	11.42	1000–90 and 88–1	0.74	Orange juice and milk	This work

^a^PEDOT—poly (3,4-ethylenedioxythiophene), ^b^PAA—polyacrylic acid, ^c^GOD—glucose oxidase enzyme, ^d^AA—anthranilic acid, ^e^PAA—polyacrylic acid, ^f^VS-PANI—vinyl substituted polyaniline, ^g^GPL-FePc—iron phthalocyanine functionalized graphene nanoplatelets, ^h^GOx—glucose oxidase, ^i^CH—hydrogel, ^j^Ag—silver, ^k^rGO—reduced graphene oxide, ^l^PdCoAg—palladium-copper-silver.

**Table 3:** Real sample and validation test for the detection of glucose using HPMpFP-graphene/GCE.

Samples	Detected (mM)	Added (mM)	Found (mM)	Recovery (%)	Glucometer (mM)	*t*-value	Critical *t*-value	*p* value
Orange juice 1	0.48 ± 0.10	0.50	0.95 ± 0.10	103 ± 2	1.9 ± 0.5	−6.15	2.92	0.033
Orange juice 2	0.43 ± 0.30	0.50	1.03 ± 0.00	90 ± 3	1.5 ± 0.6	−3.01	2.92	0.051
Orange juice 3	0.39 ± 0.20	0.50	0.87 ± 0.10	102 ± 2	1.4 ± 0.3	−6.25	2.92	0.025
Milk 1	0.21 ± 0.40	0.50	0.76 ± 0.50	93 ± 5	1.2 ± 0.4	−4.76	2.92	0.021
Milk 2	0.25 ± 0.10	0.50	0.82 ± 0.40	91 ± 4	1.1 ± 0.5	−3.01	2.92	0.049

## Data Availability

The data presented in this study are available on request from the corresponding author.
